# Automated Seizure Detection Based on State-Space Model Identification

**DOI:** 10.3390/s24061902

**Published:** 2024-03-16

**Authors:** Zhuo Wang, Michael R. Sperling, Dale Wyeth, Allon Guez

**Affiliations:** 1 Department of Electrical and Computer Engineering, Drexel University, Philadelphia, PA 19104, USA; guezal@drexel.edu; 2 Department of Neurology, Thomas Jefferson University, Philadelphia, PA 19107, USA; michael.sperling@jefferson.edu; 3 Jefferson Hospital for Neuroscience, Philadelphia, PA 19107, USA; dale.wyeth@jefferson.edu

**Keywords:** EEG, system identification, state-space model, automated seizure detection

## Abstract

In this study, we developed a machine learning model for automated seizure detection using system identification techniques on EEG recordings. System identification builds mathematical models from a time series signal and uses a small number of parameters to represent the entirety of time domain signal epochs. Such parameters were used as features for the classifiers in our study. We analyzed 69 seizure and 55 non-seizure recordings and an additional 10 continuous recordings from Thomas Jefferson University Hospital, alongside a larger dataset from the CHB-MIT database. By dividing EEGs into epochs (1 s, 2 s, 5 s, and 10 s) and employing fifth-order state-space dynamic systems for feature extraction, we tested various classifiers, with the decision tree and 1 s epochs achieving the highest performance: 96.0% accuracy, 92.7% sensitivity, and 97.6% specificity based on the Jefferson dataset. Moreover, as the epoch length increased, the accuracy dropped to 94.9%, with a decrease in sensitivity to 91.5% and specificity to 96.7%. Accuracy for the CHB-MIT dataset was 94.1%, with 87.6% sensitivity and 97.5% specificity. The subject-specific cases showed improved results, with an average of 98.3% accuracy, 97.4% sensitivity, and 98.4% specificity. The average false detection rate per hour was 0.5 ± 0.28 in the 10 continuous EEG recordings. This study suggests that using a system identification technique, specifically, state-space modeling, combined with machine learning classifiers, such as decision trees, is an effective and efficient approach to automated seizure detection.

## 1. Introduction

Epilepsy is a chronic neurological condition that affects approximately 50 million people of all ages worldwide [1]. Many patients whose seizures fail to respond to therapy undergo prolonged EEG monitoring to aid in diagnosis and plan therapy. This may be conducted in an ambulatory or inpatient setting [2]. EEG is recorded for variable periods [3,4], and these studies generate substantial data. Trained electroencephalographers must spend significant time and effort reviewing EEG to make a diagnosis, and manual review is subject to error [5]. An automatic system that detects and annotates seizures by analyzing EEG would be beneficial in reducing the time professionals must spend reviewing long-term EEG studies.

To automatically detect seizures, various detection algorithms have been proposed. Deburchgraeve et al. [6] offered an approach, tested on the recordings of 21 patients, to both analyze the correlation between high-energetic segments of EEG and detect increases in low-frequency activity with high autocorrelation, yielding 88% sensitivity and 75% positive predictive value. In another study [7] with EEG data from 17 patients, a support vector machine (SVM) was used to distinguish between seizure and non-seizure EEG epochs. The system achieved an average detection rate of 89%, with one false seizure per hour. A study employing a convolutional neural network (CNN) [8] used a training dataset of five patients and showed accuracy, specificity, and sensitivity of 88.7%, 90.0%, and 95.0%, respectively. Hassanpour et al. [9] utilized a singular value decomposition (SVD) technique to apply to a time–frequency (TF) distribution of EEG epochs and showed a 92.5% detection rate. Wang et al. [10] employed a combination of multi-domain and nonlinear features, which increased the classification accuracy to 99.3%. However, in most of these works, only a few EEG recordings were used, limiting performance evaluation.

An automated seizure detection method based on EEG state-space model identification is presented here. This method was previously shown to be effective and efficient in classifying sleep stages [11]. This work presents a preliminary evaluation of the use of machine-learning-based system identification techniques to detect seizures. We applied this method to three datasets: two provided by Thomas Jefferson University Hospital for training and testing in clinical settings, and the third was a publicly available CHB-MIT dataset [12] for cross-validation. First, we applied a system identification technique to build a mathematical model of dynamic systems of EEG epochs. Then, different orders of the dynamic system were simulated and compared with the original EEG signal. Each element of the state matrices was considered part of the features to be fed into various classifiers for training and testing. The results of this preliminary study demonstrate the potential of this proposed method to effectively detect seizures and justify further development. The use of machine-learning-based system identification techniques for seizure detection has the potential to significantly improve the accuracy and efficiency of epilepsy diagnosis, making it a valuable tool for healthcare professionals.

## 2. Material and Methods

### 2.1. Jefferson Dataset

**Figure 1:**
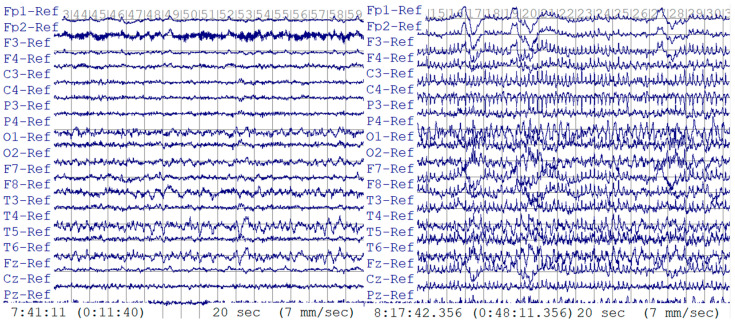
Exemplar non-seizure (**left**) and seizure (**right**) EEG data.

This retrospective study of deidentified EEG data was approved by the Thomas Jefferson University Institutional Review Board. Two datasets were provided by Thomas Jefferson University Hospital. The first dataset was used for training and testing. A total of 124 EEG recordings from 79 patients were included: 55 EEG recordings with interictal, non-seizure EEG data and 69 recordings containing seizures. EEG was recorded using international 10–20 system electrode placements plus T1 and T2 leads, and EEG was sampled at 1000 Hz. EEG recordings were visually interpreted and manually annotated by board-certified clinical neurophysiologists. The mean recording duration per EEG was 90.36 min (range, 2.8 to 180 min) for 55 non-seizure EEGs and 6 min (range, 0.7 to 20.1 min) for 69 EEGs containing seizures, for a total combined duration of 89.73 h. In Figure 1, an example of both 20 s long seizure and non-seizure EEG data is presented, illustrating the distinct differences between the two conditions. In the seizure EEG data, the characteristic pattern of seizure activity is evident through the appearance of abnormal discharges that manifest as bursts, which progressively increase in frequency, evolving into rapid, continuous spikes and waves. This distinctive pattern sets the seizure activity apart from normal, non-seizure EEG data, which does not exhibit these pronounced abnormal discharges.

We then cut the entire EEG dataset into epochs of various lengths, specifically, 1 s, 2 s, 5 s, and 10 s. The increment overlap length between any two consecutive epochs was always 50% of the epoch length. Table 1 illustrates the number of epochs generated for each epoch size.

Using the same EEG leads, the second dataset comprised ten continuous EEG recordings collected from 10 patients. Two recordings had a sampling rate of 1000 Hz, while the rest had a sampling rate of 500 Hz. The mean recording length was 24.4 h (range, 19.7 to 34.4 h), making it an extensive dataset for testing purposes. The dataset included 411 seizure periods, ranging from 16 s to 9.5 min; 1-second-long epochs with 0.5 s overlaps were used for analysis. The specificity was represented by the number of false detections per hour to evaluate the performance in clinical settings. A standard method [13] was applied where a 30 s window was considered a positive seizure detection if more than 50% of the epochs within the window were predicted as seizures. The predictions were then compared with the corresponding time segments in the annotations, and if different, it was marked as a false window. In addition, consecutive false windows were considered a single false detection.

### 2.2. CHB-MIT Dataset

This dataset was used to validate and compare the results. It was collected at Boston Children’s Hospital [14]. A total of 664 EEG recordings were collected from 23 subjects, 129 of which contained seizures. Most EEG recordings were sampled at 256 Hz using the international 10–20 system. The length and increments of each epoch were 1 s and 0.5 s, respectively. We randomly selected twenty 30 s long, non-seizure EEG segments from each subject for training. In total, we had 28,320 non-seizure EEG epochs and 14,840 seizure EEG epochs.

### 2.3. Data Preprocessing

**Figure 2:**
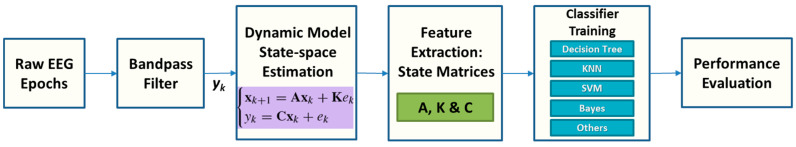
Schematic overview of the complete seizure detection algorithm.

As shown in Figure 2, the EEG dataset was initially divided into several segments with identical lengths. Next, a bandpass filter was applied to remove unwanted signals from the EEG data, such as low-frequency noise and high-frequency artifacts. Then, the dynamic systems were estimated for each bandpass-filtered EEG epoch. Finally, the state matrices of the estimated dynamic systems were extracted and used as features to train the machine learning classifiers. The state matrices were considered a compact representation of the dynamic characteristics of the EEG signal, which can capture the unique patterns of seizures. The classifiers were trained using these feature vectors, and the trained classifiers were then used to detect seizures in new EEG recordings.

**Figure 3:**
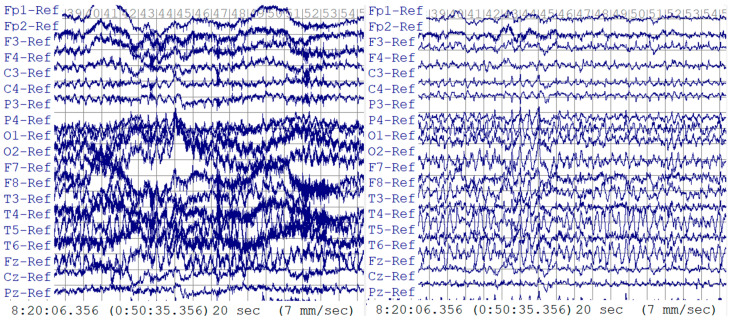
Comparison between raw (**left**) and filtered (**right**) data within a 20 s window.

**Figure 4:**
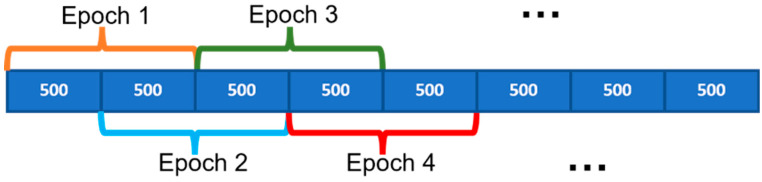
Epochs with increments.

The EEG data were filtered by a second-order Butterworth bandpass filter at 0.5–29 Hz, as this frequency range removes most unwanted signals from the EEG data, such as low-frequency noise and high-frequency artifacts, yet includes most seizure frequencies [15]. A 60 Hz notch filter was applied to remove the power line interference. The effectiveness of these filters is evident in Figure 3, which contrasts the raw and filtered EEG data. The raw data display considerable contamination from motion artifacts and power line noise, whereas the filtered data present a clearer signal in which the seizure indicators are preserved without interference. Once filtered, the data were kept as a 19×n (channel×length) matrix without time information. Each time we trained the classifier, we selected the data of a single channel from this matrix as a 1×n vector:

(1) $$Y=\left[y_{1},y_{2},y_{3},\ldots ,y_{n}\right],\;n=L,\;y_{n}\in R$$

where Y is the vector of the filtered EEG data points, $y_{n}$ is sampled at 1000 Hz, and L is the length of the vector. Using a one-second-long epoch as an example, Y was cut into epochs containing 1000 data points as 1000 Hz × 1 s. There were 500 data point increments between two consecutive epochs, as indicated in Figure 4.

Eventually, Y was resized as $Y={\left[Y_{1},Y_{2},\ldots ,Y_{p}\right]}^{T}$, described in (2).

(2) $$\left[\begin{array}{c} \begin{array}{c} Y_{1}\\ Y_{2} \end{array}\\ \vdots \\ \begin{array}{c} Y_{i}\\ \vdots \\ Y_{p} \end{array} \end{array}\right]=\left[\begin{array}{c} \begin{array}{c} y_{1}\\ y_{501} \end{array}\\ \vdots \\ \begin{array}{c} y_{500*\left(i-1\right)+1}\\ \vdots \\ y_{500*\left(p-1\right)+1} \end{array} \end{array}\;\begin{array}{c} \begin{array}{c} y_{2}\\ y_{502} \end{array}\\ \vdots \\ \begin{array}{c} y_{500*\left(i-1\right)+2}\\ \vdots \\ y_{500*\left(p-1\right)+2} \end{array} \end{array}\;\begin{array}{c} \cdots \\ \cdots \\ \cdots \end{array}\;\begin{array}{c} \begin{array}{c} y_{1000}\\ y_{1500} \end{array}\\ \vdots \\ \begin{array}{c} y_{500*(i+1)}\\ \vdots \\ y_{500*(p+1)} \end{array} \end{array}\right]$$

(3) $$p=floor\left(\frac{L}{500}\right)-1,\;i\in [1,p]$$

where $Y_{i}$ was considered the dataset for dynamic model estimation.

### 2.4. Model Estimation

This study used a system identification technique to estimate state matrices from each EEG epoch as a feature for classification. System identification uses measurements of the EEG output signal to build mathematical models of dynamic systems [16]. This means no inputs are specified since the focus is on the dynamic properties of a time series, the EEG signal [17].

There are multiple system identification approaches available, namely, autoregressive (AR), transfer function (TF), and state space (ss). We chose the state-space method, as it does not require any input to identify systems. The state-space method represents a dynamic system in terms of state and output equations, describing how the states and outputs change over time.

**Figure 5:**
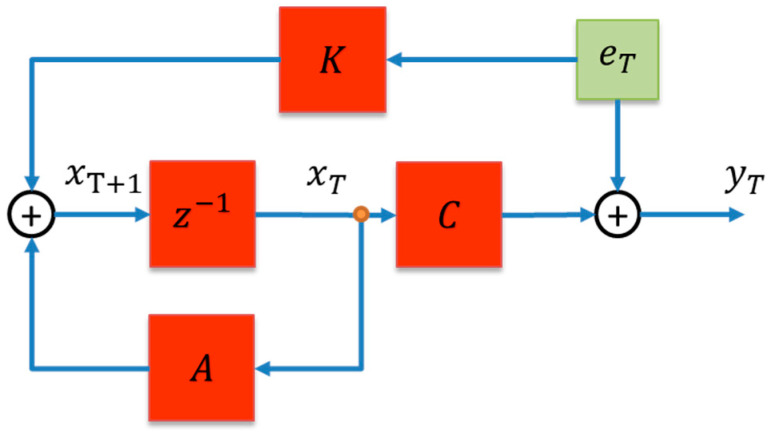
Block diagram for the proposed dynamic state-space model.

Now, consider the following discrete-time state-space dynamic system to be estimated from $Y_{i}$:

(4) $$\left\{\begin{array}{c} x_{T+1}=Ax_{T}+Bu_{T}+{Ke}_{T}\\ y_{T}=Cx_{T}+Du_{T}+e_{T} \end{array}\right.$$

where, for an $m^{th}$-order system, $y_{T}\in R$ is the output vector, and $x_{T}\in R^{m\times 1}$ is the vector of states. $A\in R^{m\times m}$ is the state transfer matrix. $B\in R^{m\times 1}$ is the input matrix. $C\in R^{1\times m}$ is the output matrix. $C\in R^{1\times 1}$ is the feedthrough matrix. $K\in R^{m\times m}$ is the steady-state Kalman gain. $e_{T}\in R$ is the zero-mean white noise. As for the input, $u_{T}$, typically, in a case like this, it should be a 1×1 scaler. However, as the output for this model is an EEG signal, also known as a time series, no input is available [17]. Thus, (4) can be written as Equation (5), also represented in Figure 5.

(5) $$\left\{\begin{array}{c} x_{T+1}=Ax_{T}+{Ke}_{T}\\ y_{T}=Cx_{T}+e_{T} \end{array}\right.$$

Without the input, $u_{T}$, the remaining state matrices, A, C, and K, of the state-space dynamic system can be estimated using the n4sid method [18]. We began by constructing a block Hankel matrix, H(k), from each EEG epoch. The Hankel matrix exhibited a unique structure, with each row shifted one time step down from the previous row.

(6) $$H(k)=\left[\begin{array}{cccc} y_{k+1} & y_{k+2} & \ldots & y_{k+q}\\ y_{k+2} & y_{k+3} & \ldots & y_{k+q+1}\\ \vdots & \vdots & \ddots & \vdots \\ y_{k+p} & y_{k+p+1} & \ldots & y_{k+q+p-1} \end{array}\right]$$

where the number of rows and columns of the Hankel matrix is p and q. p is the chosen block size, while p+q−1 equals the size of the system’s output data, which is 1000 in our case. In this study, the block size, p, was chosen to be twice the estimated system order, m, to ensure that the constructed Hankel matrix adequately captured the underlying system dynamics while avoiding unnecessary complexity. As a result, the size of the Hankel matrix is 2m×(1001−2m), and (6) can be rewritten as

(7) $$H(k)=\left[\begin{array}{cccc} y_{k+1} & y_{k+2} & \ldots & y_{k+1001-2m}\\ y_{k+2} & y_{k+3} & \ldots & y_{k+1002-2m}\\ \vdots & \vdots & \ddots & \vdots \\ y_{k+2m} & y_{k+2m+1} & \ldots & y_{1000} \end{array}\right]$$

We then applied singular value decomposition (SVD) to H(0) in order to obtain a low-rank approximation of the matrix that captures the essential system dynamics.

(8) $$H\left(0\right)=R\Sigma S^{T}$$

where H(0) is the Hankel matrix at k=0; R and S are 2m by 2m and (1001−2m) by (1001−2m) orthonormal matrices, respectively; and Σ is a 2m by (1001−2m) matrix with nonnegative numbers in the diagonal. For an $m^{th}$-order system, the ideal matrix Σ can be written as

(9) $$\Sigma =\left[\begin{array}{cc} \Sigma_{m} & 0\\ 0 & \Sigma_{*} \end{array}\right]$$

The singular values, $\Sigma_{m}\in \;R^{m\times m}$, in matrix Σ represent the importance of the corresponding singular vectors in capturing the variance of the output data. Smaller singular values or zeros in $\Sigma_{*}$ indicate that the corresponding singular vectors contribute less to the overall structure of the data. Thus, a minimal realization is obtained by eliminating $\Sigma_{*}$, and (9) can be rewritten as

(10) $$\Sigma =\left[\begin{array}{cc} \Sigma_{m} & 0\\ 0 & 0 \end{array}\right]$$

Then, we chose only the rows and columns corresponding to the $m^{th}$ model to form the matrices $R_{m}$ and $S_{m}$ and rewrite (8) as:

(11) $$H_{m}\left(0\right)=R_{m}\Sigma_{m}{S_{m}}^{T}$$

Then, the discrete-time system realization can be represented by

(12) $$A={\Sigma_{m}}^{-\frac{1}{2}}{R_{m}}^{T}H_{m}\left(1\right)S_{m}{\Sigma_{m}}^{-\frac{1}{2}}$$

(13) $$C=E^{T}R{\Sigma_{m}}^{\frac{1}{2}}$$

(14) $$K={\Sigma_{m}}^{\frac{1}{2}}{S_{m}}^{T}E$$

where $E=[I_{m},\;0]$ with I being an m×m identity matrix.

Constant m donates as the order of the system. This study estimated the models in the 3rd–10th orders. To arbitrate the most felicitous order for our study, on the one hand, we analyzed the performance of model estimation (*Fit*) that indicated similitude between the original data and the simulation of the estimated model. The *Fit* was evaluated by the normalized root mean squared error (NRMSE):

(15) $$Fit=1-100\times \frac{\sqrt{\frac{1}{1000}\sum_{T=1}^{1000}{\left(y_{T}^{sim}-y_{T}\right)}^{2}}}{\sigma (y)}$$

where $y_{T}^{sim}$ is the estimated output at time T, and $\sigma \left(y\right)$ is the standard deviation of the EEG epoch.

On the other hand, this ultimate system order was also determined by the performance of feature classification as we fed different orders of models into various classifiers.

### 2.5. Features and Classification

For each 1×1000 EEG epoch vector, $Y_{i}$, the state matrices are

(16) $$A=\left[\begin{array}{ccc} a_{11} & \cdots & a_{1m}\\ \vdots & \ddots & \vdots \\ a_{m1} & \cdots & a_{mm} \end{array}\right],\;C=\left[\begin{array}{ccc} c_{1} & \ldots & c_{m} \end{array}\right],\;K={\left[\begin{array}{ccc} k_{1} & \ldots & k_{m} \end{array}\right]}^{T}$$

The $1\times (m^{2}+2m)$ feature vector to be fed into the classifier should consist of A, C, and K, as

(17) $${feature}_{T}=\left[a_{11}\cdots \;a_{mm},\;c_{1}\cdots c_{m},\;k_{1}\cdots k_{m}\;\right]$$

For each EEG recording, there were p feature vectors, as described in (2). For example, the Jefferson dataset contained 149,595 feature vectors, of which 49,595 were labeled as seizures. The label of each feature vector was made according to the annotation provided by the expert viewers. There were only two classes, “1” for seizure and “0” for non-seizure.

Sensitivity, specificity, and accuracy were used as the performance statistics. These terms can be determined by the “Standard of Truth” [19] as true positive (*TP*), true negative (*TN*), false negative (*FN*), and false positive (*FP*) (18)–(20).

(18) $$Sensitivity=\frac{TP}{TP+FN}$$

(19) $$Specificity=\frac{TN}{TN+FP}$$

(20) $$Accuracy=\frac{TP+TN}{TP+TN+FP+FN}$$

Both *TP* and *TN* indicate consistency between the annotation and classifier, while *FP* and *FN* suggest contradiction. In this study, we defined seizure as positive and non-seizure as negative. Therefore, the sensitivity and specificity represent the degree of excellence of a classifier in identifying seizures and non-seizures, respectively. And the accuracy is the proportion of correct labels throughout the entire dataset.

All three statistics were verified by the 10-fold cross-validation method. The EEG epochs were first shuffled randomly and divided into ten equal portions. Then, 90% of the EEG epochs were used as a training dataset, while the rest, 10%, were for testing. This cross-validation process was repeated ten times, with each of the ten portions used once as the validation dataset. The final evaluation statistics reported in this study were the average value obtained from the above ten tests.

To validate the efficacy of our proposed model and feature set for automated seizure detection, this study selected kernel naïve Bayes, linear discriminant, linear SVM, fine KNN, and bagged trees as the candidate classifiers. By employing a range of basic, yet diverse, classifiers, we aimed to test our model’s robustness and generalizability across different algorithmic approaches. We trained all the classifiers with features converted from 3rd- to 10th-order models. The comparison was made across different classifiers and orders to designate the most suitable dual. Once the system order and classifier were settled, we performed the validation by applying our method to a publicly available dataset: CHB-MIT.

## 3. Results

### 3.1. Model Estimation

State-space models of orders 3 to 10 were estimated for each EEG epoch, and the model order, m, was chosen based on a trade-off between the feature’s size and the classifier’s performance. Choosing the appropriate model order is vital for an automated seizure detection system, as it can affect the system’s performance. The higher the order, the more complex the model, which can lead to overfitting. On the other hand, if the order is too low, the model may not be able to capture the essential dynamics of the EEG signal. The feature size is the number of parameters used to represent the EEG signal and was provided in (17) as $1\times (m^{2}+2m)$. Therefore, the selected order should be as small as possible to minimize the training cost while maintaining a reasonable Fit accuracy and a decent classifying performance.

The average Fit accuracy was relatively steady at 98.3% ± 0.11% across the selection of model orders from 3 to 10. The decision on the model order was solely based on the classifier’s performance rather than the fit accuracy of the state-space model. By selecting a more petite model order, the feature size will be smaller, reducing the computational cost of the classifier and allowing for faster training and prediction.

### 3.2. Classification

We have chosen kernel naïve Bayes, linear discriminant, SVM, KNN, and decision trees as the classifiers to train our features. These basic classifiers consume less computing time and resources to train and validate than deep learning, as we hope to find that the system identification method can effectively and efficiently detect seizures. By feeding 1 s epoch feature vectors of the 3rd- to 10th-order systems into the classifiers, we could then evaluate the performance of different model orders and determine which order is most appropriate for our application.

**Figure 6:**
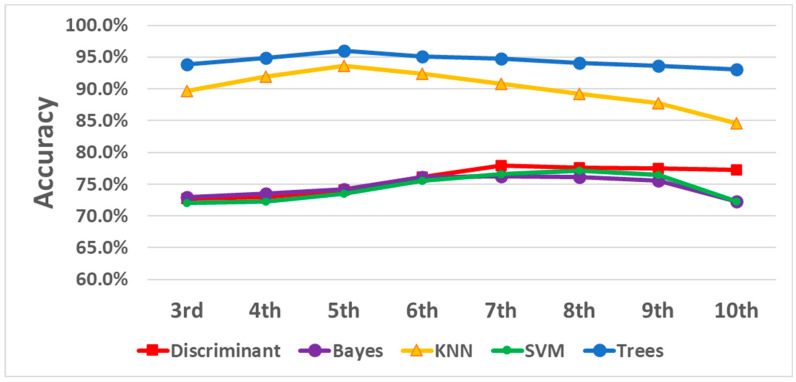
Classifier accuracy over systems of different orders.

The results are illustrated in Figure 6 and Table 2. Correlations between the growth of system order and validation accuracy were not consistent among different classifiers. The KNN and decision trees started with an escalation trend and then reached a peak turning point at the fifth order. The kernel naïve Bayes, linear discriminant, and SVM increased accuracy as the system order increased from 3 to 9 and then declined in performance at order 10. Moreover, decision trees outperformed the rest of the classifiers with the highest standard over the systems of every order. KNN caught up with the others at the fifth order with a 93.6% accuracy. Eventually, with the fifth-order system, the trees provided the highest accuracy, 96.0%, with a sensitivity of 92.7% and a specificity of 97.6%. These results indicate that the decision trees classifier was better suited for this analysis and that choosing the fifth model order balanced complexity and performance.

We also estimated the fifth-order system of epochs in different lengths, such as 2 s, 5 s, and 10 s, in addition to 1 s long epochs. The decision tree classifier then trained the feature vectors obtained from these epochs. The results of the different epoch lengths are listed and compared in Table 3 to show the effect of the length of the epochs on the system’s performance. As is seen in the table, performance metrics decreased as the epoch length increased. Specifically, accuracy dropped from 96.0% to 94.9%, sensitivity fell from 92.7% to 91.5%, and specificity sank from 97.6% to 96.7% as the epoch length grew from 1 to 10 s. These results indicate that the choice of epoch length impacts the performance of the seizure detection system.

**Figure 7:**
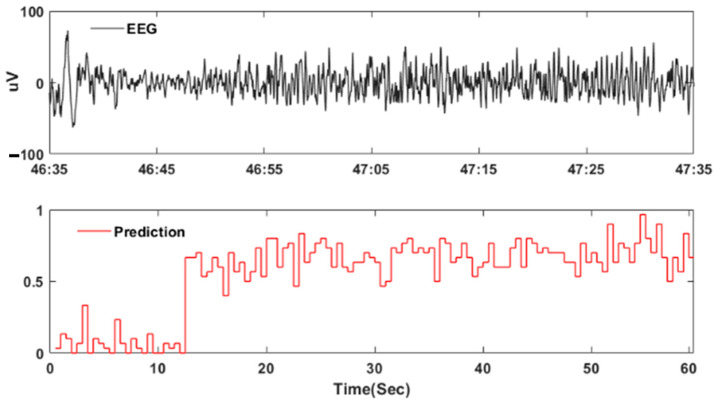
Exemplar of seizure EEG and its prediction score.

The analysis of the ten continuous EEG recordings showed that, on average, the system produced 0.5 false detections per hour of the EEG recording, with a margin of error of ±0.28 false detections per hour. In addition, by using a 30 s decision window and 1 s long epochs with 0.5 s overlaps, the method could detect all seizures within the first feasible window, with no additional delay other than the minimum required 15 s within each 30 s window to make the decision. Figure 7 shows a continuous EEG recording lasting one minute. During this recording, a seizure began at 46 min and 47 s. Our detection method accurately identified this seizure. More information about the overall results can be found in Table 4.

To validate the proposed method and conduct a further assessment, we also applied this method to the CHB-MIT dataset. This method achieved 94.1% accuracy, with 87.6% sensitivity and 97.5% specificity. Hence, our proposed method works on an independent dataset. Additionally, we observed that these results were not subject-specific but generalized over the entire dataset. Table 5 illustrates a comparison of the performance results of other studies that also worked on the whole CHB-MIT database. The results show that the proposed method outperforms other studies regarding accuracy and specificity. Sensitivity was better than all but one method.

## 4. Discussion

The present analysis shows that state-space modeling, combined with a decision tree machine learning classifier, is an effective approach to automated seizure detection. We achieved good sensitivity and specificity, as well as accuracy. However, accuracy cannot fully represent the actual performance of a detector. The reason is that most epochs are non-seizure epochs for any EEG recording. For example, a 90% accuracy could be generated by 99% specificity and 50% sensitivity if 80% of the dataset is negative. Hence, the problem at hand is complex. For clinical utility, both high sensitivity and specificity are required. Sensitivity is most important, as failure to detect seizures makes any detection system less useful. Efficiency is achieved with high specificity, minimizing the time needed for human review. The method described in this paper yielded superior accuracy and specificity compared with most other reports. Our method better-identified epochs without seizures and was as good at detecting seizures with high accuracy. This result is acceptable for preliminary work but may be insufficient for clinical needs and needs further improvement, as each patient can yield a unique EEG wavelet pattern [25] due to differing seizure types, and some patients have multiple seizure types. Therefore, if we model and train on individual EEGs yet characterize them as similar, the detection rate is undoubtedly decreased.

There are other studies that have focused on a “case-by-case” situation. For example, Zabihi et al. [26] performed a subject-specific study with an average of 93.7% sensitivity and a specificity of 99.05% in four subjects. Another study [27] proposed a patient-dependent system with 97.12% specificity and 99.29% sensitivity. Alternatively, an individual classifier can be built for each channel and seizure pattern [28], eventually reaching an average accuracy of 95.12%. Similarly, for a subject-basis analysis, our method generated an average of 98.3% accuracy, 97.4% sensitivity, and 98.4% specificity. However, this level of performance has only been shown post hoc, and the clinical problem of seizure detection in large numbers of patients is not suited to this approach. Such a precisely customized classifier would only prove useful if individualized preliminary data were available for training. Therefore, both comprehensive dataset-based and customized classifiers can be useful, though the former are best suited when an unknown EEG dataset must be analyzed. A classifier pre-trained on a full-scale dataset validates itself in robustness, adaptation, and performance. A customized classifier would likely be better with delayed deployment after onsite training.

Our method demonstrated its clinical potential through a dataset of 10 consecutive EEG recordings. By using a 30 s decision window and 1 s long epochs with 0.5 s overlap, the system could accurately detect all seizures within the first feasible window. The low false detection rate confirms the system’s effectiveness. On the other hand, the sizeable false detection range indicated the variability in the false detection rate across the ten patients. Several solutions might address this issue, including improving the signal quality by implementing advanced noise reduction techniques or re-evaluating the decision threshold to account for the impact of noise and artifacts on the data. Additionally, extra data sources, such as clinical history, demographic information, or behavioral data, could be integrated into the model to reduce the false detection rate.

Additional work is required. The proposed method is still in the early stages of development, and there is room for improvement. Our study was restricted to the analysis of one-second epochs. A one-size-fits-all approach may not be appropriate. However, as seen in Table 3, increasing epoch size is associated with decreased detection sensitivity. One possible reason for this is that using longer epochs reduces the number of available segments for training decreases. This negatively impacted the classifier’s overall performance. Also, the length of the EEG epoch can affect the sensitivity and specificity of seizure detection methods. Shorter epochs can increase the temporal resolution but may miss ongoing seizures with ictal patterns that last longer than the epoch length [29]. On the other hand, longer epochs may provide a better view of prolonged seizures and increase the chance of missing short seizures that occur within these longer epochs. Therefore, it is essential to carefully consider the trade-offs and choose an appropriate epoch length for the specific application and dataset.

One of our proposed method’s limitations is that it does not specify the seizure types. Defining seizure types may allow the classifiers to be trained to recognize specific seizure types and improve the method’s specificity. However, it is important to acknowledge that this hypothesis must be tested and validated through further research and experimentation to determine its actual impact on the model’s specificity. Another area for improvement is the method’s robustness in dealing with artifacts, as artifacts can significantly affect accuracy. Deep learning techniques, such as convolutional neural networks (CNNs), have shown promise in identifying and removing artifacts from EEG signals [30]. These techniques could increase the proposed method’s robustness and performance.

## 5. Conclusions

The proposed system leverages a state-space-model-based system identification method for automated seizure detection in EEG recordings. By processing EEG time series signals, this method constructs mathematical models to efficiently represent signal epochs with a minimal set of parameters. These parameters, utilized as features for machine learning classifiers, demonstrated the efficacy of combining a fifth-order dynamic system with a decision tree classifier. An evaluation of this approach using the Jefferson and CHB-MIT datasets yielded high accuracies and excellent specificities while also indicating that sensitivity has the potential for enhancement. Adjusting the classifier on an individual basis substantially improved sensitivity and accuracy, underscoring the effectiveness of personalized detection strategies. The proposed method also demonstrated its potential in a clinical setting through this dataset of ten consecutive EEG recordings. Future developments could focus on integrating larger and more diverse datasets alongside advanced deep learning classifiers to broaden the system’s capability in identifying various seizure types with increased sensitivity. This work lays the foundation for more automated, accurate, and efficient seizure diagnosis, promising to augment clinical practice and patient outcomes.

## Figures and Tables

**Table 1:** Number of epochs generated for each epoch length.

Epoch Size	1 s	2 s	5 s	10 s
Seizure Epochs	49,595	24,386	9837	4866
Non-Seizure Epochs	100,000	49,611	19,182	9340
Total Epochs	149,595	73,997	29,019	14,206

**Table 2:** Classifier accuracy over systems of different orders.

	System Orders	3rd	4th	5th	6th	7th	8th	9th	10th
Classifiers									
Discriminant		72.7%	73.0%	74.1%	76.1%	77.9%	77.6%	77.5%	77.3%
Bayes		72.9%	73.5%	74.2%	76.1%	76.2%	76.1%	75.5%	72.3%
KNN		89.7%	91.9%	93.6%	92.4%	90.8%	89.2%	87.8%	84.6%
SVM		72.0%	72.3%	73.5%	75.6%	76.6%	77.1%	76.5%	72.3%
Trees		93.8%	94.9%	96.0%	95.1%	94.8%	94.1%	93.6%	93.1%

**Table 3:** Training results for different-length epochs.

Epoch Size	Increment	Sensitivity	Specificity	Accuracy
1 s	0.5 s	92.7%	97.6%	96.0%
2 s	1 s	92.6%	97.4%	95.8%
5 s	2.5 s	92.3%	97.0%	95.4%
10 s	5 s	91.5%	96.7%	94.9%

**Table 4:** Detail of 10 continuous EEG records.

Subject	EEG Duration (h)	Number of Seizures	Seizure Length (s)	Total False Detections	False Detections per Hour
1	24	1	576	5	0.2
2	24	7	43~104	10	0.4
3	21.3	44	30~310	18	0.8
4	24.1	114	30~49	28	1.2
5	24	13	30~120	10	0.4
6	19.7	120	16~120	10	0.5
7	24	1	122	6	0.3
8	24.1	72	19~180	12	0.5
9	24.8	33	37~77	10	0.4
10	34.4	6	58~297	9	0.3
Total	244.4	411		118	
Average	24.44	41.1		11.8	0.5
STDEV.	3.6	43.6		6.3	0.28

**Table 5:** Performance comparison of studies on whole CHB-MIT datasets.

Method	Accuracy	Sensitivity	Specificity
Khan [20]	91.8%	83.6%	100%
Gill [21]	86.93%	86.26%	87.58%
Lima [22]	88.45%	85.59%	91.32%
Birjandtalab [23]	-	80.87%	47.45%
Fergus [24]	-	84%	85%
This Work	94.1%	87.6%	97.5%

## Data Availability

The data supporting this study’s findings are available from the corresponding authors upon reasonable request. The data are not publicly available due to the data sharing restriction from the data provider.
